# On the effective stress law for rock-on-rock frictional sliding, and fault slip triggered by means of fluid injection

**DOI:** 10.1098/rsta.2016.0001

**Published:** 2017-08-21

**Authors:** Ernest Rutter, Abigail Hackston

**Affiliations:** Rock Deformation Laboratory, School of Earth and Environmental Sciences, University of Manchester, Manchester M13 9PL, UK

**Keywords:** fluid injection, induced fault slip, aseismic and seismogenic, experimental rock mechanics

## Abstract

Fluid injection into rocks is increasingly used for energy extraction and for fluid wastes disposal, and can trigger/induce small- to medium-scale seismicity. Fluctuations in pore fluid pressure may also be associated with natural seismicity. The energy release in anthropogenically induced seismicity is sensitive to amount and pressure of fluid injected, through the way that seismic moment release is related to slipped area, and is strongly affected by the hydraulic conductance of the faulted rock mass. Bearing in mind the scaling issues that apply, fluid injection-driven fault motion can be studied on laboratory-sized samples. Here, we investigate both stable and unstable induced fault slip on pre-cut planar surfaces in Darley Dale and Pennant sandstones, with or without granular gouge. They display contrasting permeabilities, differing by a factor of 10^5^, but mineralogies are broadly comparable. In permeable Darley Dale sandstone, fluid can access the fault plane through the rock matrix and the effective stress law is followed closely. Pore pressure change shifts the whole Mohr circle laterally. In tight Pennant sandstone, fluid only injects into the fault plane itself; stress state in the rock matrix is unaffected. Sudden access by overpressured fluid to the fault plane via hydrofracture causes seismogenic fault slips.

This article is part of the themed issue ‘Faulting, friction and weakening: from slow to fast motion’.

## 1. Introduction

Fluid injection into deep rocks is increasingly used in connection with energy extraction [1–5]. Examples include: re-injection for maintenance of reservoir stability, hydraulic fracture for well stimulation and unconventional hydrocarbon production, and deep geothermal energy production whether or not involving hydraulic fracture. Deep injection is also widely used for disposal of fluid wastes from manufacturing or of well flowback fluids, and includes injection for underground gas disposal (e.g. CO_2_) and storage (e.g. natural gas).

Deep fluid injection can raise the pore pressure in hydraulically conductive rocks, or injection into impermeable rocks can raise the fluid pressure in natural faults and fissures [6]. In these cases, there is potential to promote fault slip. Since the anthropogenically induced tremors at Denver in the 1960s [7], it has been clear that this can lead to induced or triggered seismicity [1–3,5,6,8–15]. However, it must not be assumed that fault slip induced by fluid injection is necessarily seismogenic; it can be stable. The energy release and hence magnitude of such seismicity is strongly sensitive to the amount, rate and pressure of fluid injected, as a result of the way that seismic moment release is related to slipped area [6,10]. McGarr *et al*. [11] demonstrated that the size of induced earthquakes scales with the amount of fluid injected and that management of fluid injection was the key to minimizing the hazard of induced earthquakes.

The hazards of seismicity induced through fluid injection have been widely studied through *in situ* monitoring [3,9,16,17] and numerical modelling, but the effects can also be demonstrated on a small scale through laboratory experiments at high pressures, and through mesoscale injection experiments into a natural fault in an underground laboratory [18]. Here, we investigate both stable and unstable induced fault slip on pre-cut planar surfaces in two sandstones, Darley Dale sandstone and Pennant sandstone. The rocks were chosen for their very different permeabilities to fluid flow, while their mineralogies are broadly comparable. After establishing the basic frictional properties of these rocks, we investigate their response to fluid injection while under shear stress and normal stress combinations below those normally required to instigate frictional sliding.

## 2. Sample characterization and experiments performed

Two quartz sandstones of different porosities and permeabilities were used:

(a) Pennant sandstone, a grey, durable quartz sandstone of upper Carboniferous age from South Wales [19]. Modal composition was obtained by chemical mapping on the scanning electron microscope (SEM), and porosity was determined by gravimetry and by helium porosimetry (table 1).
(b) Darley Dale sandstone, an upper Carboniferous quartz sandstone from Derbyshire, England. This decorative stone has previously been widely used in rock mechanics investigations [20–23].

**Table 1.** Modal composition and key parameters of the two sandstones studied. UCS, unconfined compressive strength; *τ*_o_, cohesive strength ± values at 1 s.d. qtz, quartz; fsp, feldspar; musc, muscovite.

rock type	mineralogy	porosity	grain size (μm)	UCS (MPa)	*τ*_o_ (MPa)	friction coeff.
Pennant	qtz 70%, fsp 15%, interstitial musc + oxides + clays 10%	0.046 ± 0.002	200 ± 90	154.0 ± 14.2	28.4 ± 3.6	0.66 ± 0.01
Darley Dale	qtz 67%, fsp 16%, mica + clay 3%	0.135 ± 0.01	400 ± 250	85.0 ± 6.9	22.0 ± 1.9	0.65 ± 0.01

**Figure 1.**
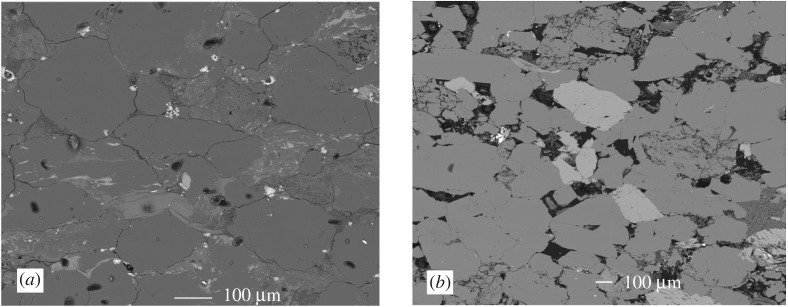
Backscattered electron images showing microstructure of (*a*) Pennant and (*b*) Darley Dale sandstones. Bedding trace is horizontal in both cases. Both rocks are dominated by quartz grains (mid-grey), and there is some feldspar (light grey) in Darley Dale sandstone. Clay and muscovite partially fill interstitial spaces in Darley Dale sandstone and wholly in Pennant sandstone. A weak grain shape fabric (grain long axes horizontal) exists in the Pennant sandstone. Porosity appears black.

Both rocks display little evidence of heterogeneity and anisotropy. Their typical microstructures are shown as SEM backscattered electron images in figure 1. Both rocks display a weak grain shape fabric parallel to bedding orientation. Their different permeabilities *k* (m^2^) are central to this investigation, and they differ by a factor of approximately 10^5^.

Permeability of Darley Dale sandstone to water, normal to bedding, was reported by Zhu & Wong [20] and is sensitive to Terzaghi effective confining pressure *Pe* (MPa; defined as confining pressure − pore pressure), given by

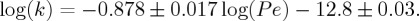


**Figure 2.**
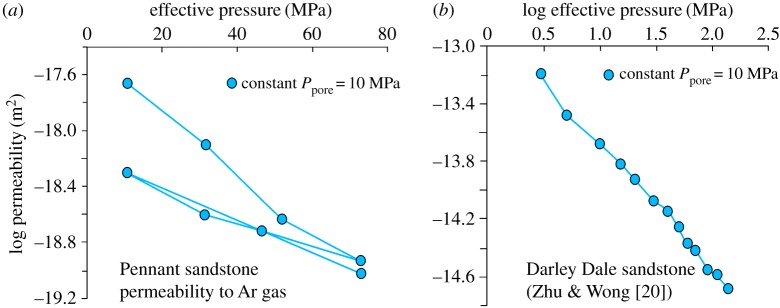
Permeability (*k*) data for (*a*) Pennant (gas pore fluid) and (*b*) Darley Dale (water pore fluid) sandstones. After the first pressure cycle Pennant sandstone is described by log *k* = −0.0113 ± 0.001*Pe* −18.2 ± 0.05 and Darley Dale sandstone by log(*k*) = −0.878 ± 0.017 log (*Pe*) – 12.8 ± 0.03. Errors of measurement are generally smaller than errors of fit, and commensurate with plotted point size. (Online version in colour.)

Application of 70 MPa effective pressure decreases permeability from 10^−13.2^ m^2^ at 3 MPa effective pressure to 10^−14.6^ m^2^ (figure 2*b*). The permeability of Pennant sandstone to argon gas was measured normal to bedding using the oscillating pore pressure method [24] over the effective pressure range 1–70 MPa and is much lower than that of Darley Dale sandstone. It may be described by

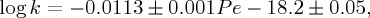

thus permeability decreases from about 10^−18.2^ to 10^−19^ m^2^ over the same effective pressure range (figure 2*a*). Effective pressure change produced by varying pore pressure at constant confining pressure has the same effect on permeability as changing confining pressure at constant pore pressure. The permeability of this rock is comparable to that of many shales [25]. It is a tight sandstone. This is an important consideration when pore fluid pressure is applied.

Preliminary measurement [26] of the hydraulic conductivity *kt* (m^3^) of the sawcut interface for Pennant sandstone follows the law

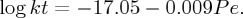


*Pe* is equivalent to normal stress across the interface. To compare, note that matrix permeability is equivalent to the hydraulic conductivity of a layer 1 m thick. Thus, an interface of nominal 10 µm thickness (*t*) under 1 MPa normal stress behaves as if its permeability (*k*) were 10^6^× greater than the bulk permeability, with the contrast becoming greater at higher hydrostatic pressure. Owing to its greater bulk permeability we have no data for the hydraulic conductivity of a planar interface in Darley Dale sandstone, but we can expect it to be comparable to Pennant sandstone. In this case, the ‘permeability’ of a planar crack will be comparable with that for flow through the rock matrix, i.e. a crack conveys no conductive advantage in Darley Dale sandstone.

**Figure 3.**
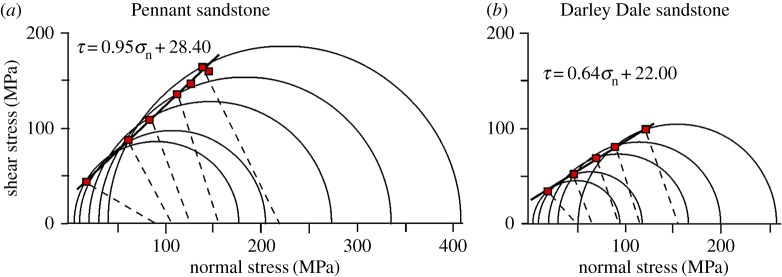
Ultimate strength of (*a*) Pennant and (*b*) Darley Dale sandstones under axisymmetric compressive loading expressed as Mohr circles at failure. Also shown are resolved normal and shear stresses at the point of failure on the fault planes that formed, the orientations of which are half of the angle subtended by the dashed lines with the abscissa. The Mohr envelopes are not shown, but they would lie at slightly higher stresses than the best fits to the resolved stresses on the fault planes that formed. Except at low pressure, fault angles are systematically larger than would be predicted by the simple Mohr–Coulomb criterion. Uncertainties in fits are shown in table 1. Errors of measurement are generally smaller than plotted point size. These data were previously reported in Hackston & Rutter [23]. (Online version in colour.)

**Figure 4.**
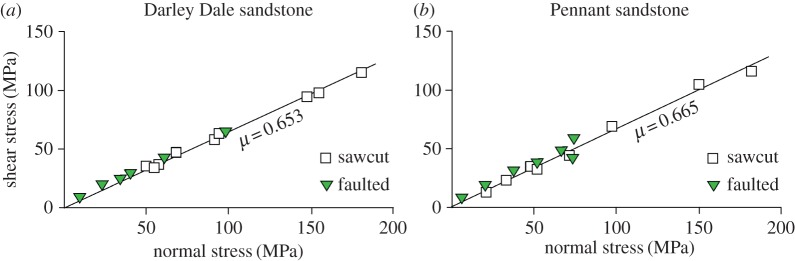
Frictional sliding data on sawcuts loaded in axisymmetric compression (including sawcut angles 35°, 45° and 55° to the cylinder axis of the sample) and some freshly faulted surfaces for (*a*) Darley Dale and (*b*) Pennant sandstones. Uncertainties in fits are shown in table 1. These data were previously reported in Hackston & Rutter [23]. (Online version in colour.)

The influence of effective confining pressure on the strength of these rocks was reported by Hackston & Rutter [23]. Samples were tested oven (60°C) dry under axisymmetric shortening (conventional triaxial) loading conditions (figure 3). The porosity differences have a major impact on the relative unconfined and cohesive strengths of these rocks (table 1). The coefficient of frictional sliding was measured both on freshly produced fault surfaces and on planar sawcut surfaces (figure 4).

They are almost identical (0.66 and 0.65, respectively, for Pennant and Darley Dale sandstones) and correspond with the generalization of Byerlee [27,28] that friction in rocks is, to a useful approximation, almost independent of rock type. In Hackston & Rutter's [23] experiments, these rocks displayed stable sliding on fault and sawcut surfaces under the full range of conditions imposed, both with and without static pore fluid pressures applied.

## 3. Experimental methods employed

All experiments in this study were carried out on 20 mm diameter × 45 mm nominal length cylindrical samples cored normal to bedding orientation. Cylinders were sawcut at an angle of either 35°, 45° or 55° to the cylinder axis and the cut surfaces were ground using a 60 µm grinding wheel and lightly finished using 16 µm abrasive paper. Prepared samples were stored in an oven at 60°C until used. Samples were jacketed with an inner sleeve 2 mm thick of silicone rubber and an outer jacket of heat-shrink rubber. The purpose of the inner jacket was to inhibit perforation of the outer, sealing jacket by the sharp edge of the sawcut sample half during slip events. The jackets were established not to contribute any significant load-bearing capacity to the sample assembly (0.1 MPa or less). All samples were shortened parallel to the cylinder axis. Loading stress paths included deformation both at constant confining pressure (smallest principal stress *σ*_3_ = *σ*_2_) and at constant resolved normal stress across the slip plane, maintained by servo-control of the confining pressure (table 2). The confining pressure fluid was a synthetic ester, dioctyl sebacate (Reolube DOS^®^), a linear viscous fluid whose viscosity at atmospheric pressure is 0.024 Pa s, about 24 times that of water. Compared with common mineral oils, it displays only a small viscosity increase over the 100 MPa pressure range used in this study. Compressibility was measured to be 4.71 × 10^−10^ Pa^−1^, insensitive to pressure above 50 MPa. The same fluid was used for pore fluid pressure when a relatively viscous fluid was required. In other tests, argon gas was used as pore fluid. It has a viscosity of approximately 6 × 10^−4^ Pa s at 50 MPa pressure and 20°C [29]. Compressibility is nearly insensitive to pressure up to about 150 MPa, beyond which it decreases by 60% by 100 MPa pressure. Water was avoided as a pore fluid to eliminate possible water weakening effects relative to tests on the dry rock, such as have been previously reported for some sandstones [30–32].

**Table 2.** Summary of fluid injection tests performed and experimental conditions. All tests start at 69 MPa confining pressure except DDA24 and DDA25 (100 MPa), and DDA6 (50 MPa). Tests are grouped according to cases (a) through (d) described in the text.

test no.	rock type	pore fluid	gouge	sawcut angle	stress path	sliding	comment
case (a) conductive rock matrix							
DDA24	Darley Dale	oil	yes	35°	const. *Pc*	stable	*Pp* effective
DDA25	Darley Dale	oil	yes	45°	const. *Pc*	stable	*Pp* effective
DDN3	Darley Dale	oil	yes	55°	const. *Pc*	stable	new fault @ 34°, *Pp* effective
DDA6	Darley Dale	none	no	55°	const. *Pc*	stable	new fault @ 35°
case (b) non-conductive rock matrix, conductive fault							
Pen15	Pennant	oil	yes	45°	const. *Pc*	stable	*Pp* effective
Pa2a3	Pennant	oil	yes	45°	const. *σ*_n_	stable	*Pp* effective
Pen20	Pennant	oil	yes	35°	const. *Pc*	stable	*Pp* effective
Pa8	Pennant	oil	yes	55°	const. *Pc*	stable	*Pp* effective
Pa3a1	Pennant	gas	no	45°	const. *σ*_n_	stable	*Pp* effective
Pa3a2	Pennant	gas	no	45°	const. *Pc*	stable	*Pp* effective
Pa2a3	Pennant	gas	no	45°	const. *σ*_n_	stable	*Pp* effective
Pa2a4	Pennant	gas	yes	45°	const. *Pc*	stable	*Pp* effective
case (c) non-conductive rock matrix, non-conductive fault							
Pa4a	Pennant	oil	no	45°	const. *Pc*	stable	overpressure required
Pa4b	Pennant	oil	no	45°	const. *Pc*	stable	overpressure required
case (d) non-conductive rock matrix, conductive fault, hydraulic fracture required							
Pa7	Pennant	oil	yes	45°	const. *Pc*	stick–slip hydrofracture	oil
Pa9	Pennant	oil	yes	55°	const. *Pc*	stick–slip hydrofracture	oil

Axial load was measured using an internal load cell that permitted stress measurements to an accuracy of better than 0.5 MPa. Axial loading and confining pressure regulation was achieved by computer-controlled electromechanical servo-systems. Confining and pore fluid pressures were measured to an accuracy of better than 0.3 MPa. Axial displacement is measured outside the pressure vessel, and specimen displacements were determined by subtracting the calculated elastic machine displacement from the total measured displacement using an apparatus stiffness of 82 kN mm^−1^. All loading increments were made at a total axial displacement rate of 8.0 × 10^−4^ mm s^−1^

**Figure 5.**
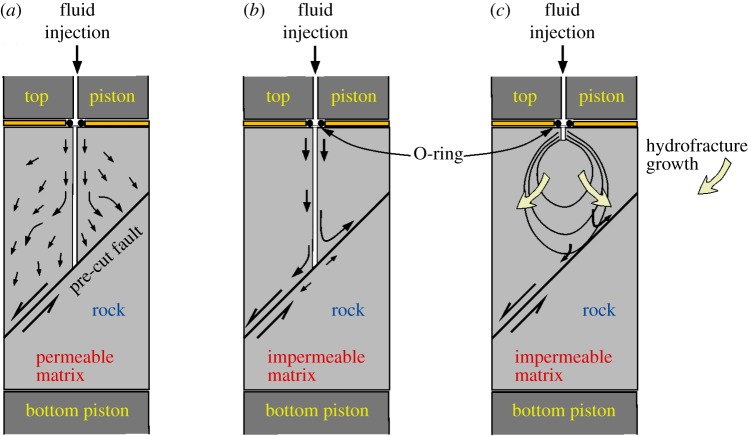
Schematic of the three specimen configurations employed: (*a*) Darley Dale sandstone and (*b*) and (*c*) Pennant sandstone. (Online version in colour.)

The aim of the experiments was to inject pressurized fluid into rock samples containing pre-stressed fault planes and hence to induce fault slip. Both high- and low-viscosity fluids were used (oil and argon gas) and fault planes were made less or more hydraulically conductive either by leaving them clear and close-fitting, or by introducing a 0.5 mm thick layer of fault ‘gouge’ in the form of granulated quartz with 75% of grains in the size range 38–63 µm. For most experiments pore fluid was supplied directly to the centre of the fault plane via a 1.0 mm diameter hole drilled through the upper forcing block. The external pore fluid pump was connected to the sample via a hole drilled down the centre of the upper loading piston (figure 5). At the junction between the hollow loading piston and the central hole in the rock sample, an ‘O’-ring seal was used to prevent leak-off of pore fluid to the interface between the outer rubber jacket and the specimen, and hence to permit pore fluid overpressure relative to the confining pressure.

For the typical 1 h duration of an experiment, this was the only access route for pore fluid (whether liquid or gas) to the fault plane in the case of the low-permeability Pennant sandstone. For the much higher-permeability Darley Dale sandstone, the pore fluid could communicate with the whole area of the fault plane by flow through the permeable matrix of grains, in addition to the route via the central hole. This difference between the two rocks has a profound effect on mechanical behaviour during fluid injection.

In the case of Pennant sandstone, experiments were also carried out in which the connection from the pore fluid pipe in the loading piston was made through the upper forcing block to the fault plane by means of a hydraulic fracture. This required a substantial fluid overpressure to develop in the pore pressure system that was suddenly released into the fault plane when the hydrofracture eventually formed and penetrated to the fault plane. To initiate the hydrofracture, a shallow hole, 4 mm long, was drilled into the upper forcing block (figure 5*c*). The end of the hole could be pressurized well in excess of the applied confining pressure, until leak-off (hydraulic fracture initiation) and then shear failure (differential stress drop) occurred.

Slip on an inclined sawcut in the ‘triaxial’ testing configuration is not perfect. Substantial shear offsets can change the cross-sectional area of contact between the specimen halves, and the lateral displacement of the axial column tends to modify the stress state in the sample as a result of induced bending forces [33]. These effects can be minimized by keeping inelastic shear offsets small, less than 3 mm. Further, the apparatus used is able to deform samples in axial extension as well as in axial shortening. This allows a sheared sample to be reverse-sheared to restore the alignment of the forcing blocks. It was found that re-shearing the same sample did not significantly affect the frictional behaviour [23].

Faults were pre-stressed to combinations of resolved shear and normal stress corresponding to the onset of slip, over a time period of 30 min but without any pore pressure. Then pore pressure was increased at a uniform rate until slip began (e.g. after about 3 min). Slip on the sawcut surfaces, whether or not gouge was present, was indicated after the test by the presence of slip striations on the faults. The progression of slip as pore pressure was increased caused the differential stress to decrease, i.e. the diameter of the Mohr circle representing the stress state decreased, while the total confining pressure was maintained constant. This caused the stress condition (combination of resolved shear stress and normal stress) on the fault to decrease below the sliding condition, but sliding was maintained by continually increasing the pore pressure, by pumping pore fluid into the specimen.

To summarize, three different test configurations, (a), (b) and (c), were investigated, and these are illustrated in figure 5.

(a) Oil or gas was injected into permeable Darley Dale sandstone. The fluid could access the whole fault area via the permeable matrix.
(b) Fluid was injected directly into the fault plane via a hole drilled through the top half of the specimen of low-permeability Pennant sandstone. Hydraulic conductivity of the fault plane could be varied by testing with or without a granular gouge layer in the fault plane.
(c) Fluid (oil) access to the fault could occur only after completion of a hydraulic fracture linking the fluid injection point to the fault plane, in low-permeability Pennant sandstone.

## 4. Experimental results

For each of the test configurations employed, we present an anticipated result followed by actual experimental observations. Details of experiments performed are shown in table 2. In each case the expected friction sliding criterion is as shown in figure 4, and confining pressure used was held constant at 69 MPa as pore fluid was injected, rising at a constant rate of change of pressure of nominally 5 × 10^−2 ^MPa s^−1^, until the sample was fully offloaded after a period of about 30 min. Volume rate of injection thus varies according to pressure and fluid compressibility, and the storage capacity of the rock sample and pipework.

The relative magnitudes of permeability of the rock matrix, the hydraulic conductivity of the fault plane and the viscosity of the infiltrating fluid are expected to determine mechanical behaviour of the rock/fluid system. Depending on their values, different things (cases (a) through (d) below) can be expected to happen.

### (a) Case of injection into a rock of high permeability

**Figure 6.**
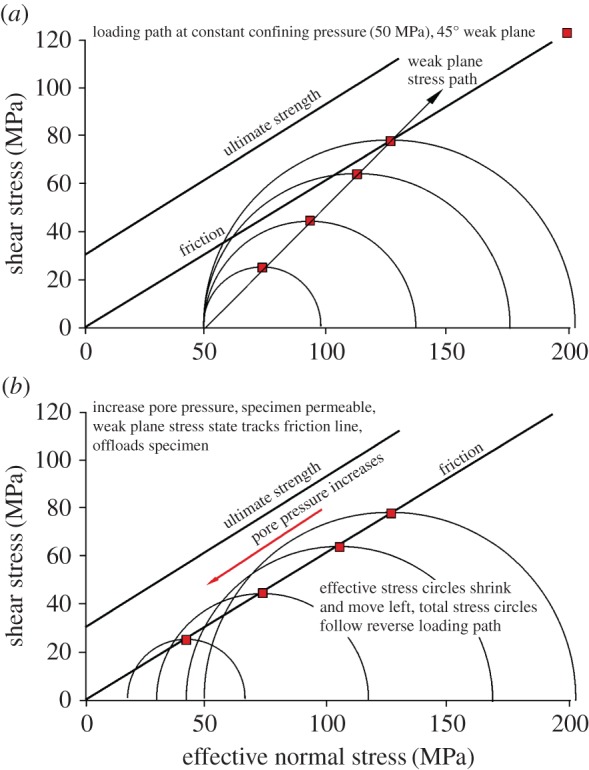
Predicted history of a test on Darley Dale (permeable) sandstone with a 45° pre-cut at a constant total confining pressure = 50 MPa. (*a*) The loading path (shear and normal stress) on the pre-cut, as a series of expanding Mohr circles with the minimum principal stress held constant at 50 MPa. (*b*) How progressively raising the pore pressure shifts the Mohr circle leftward, allowing progressive offloading by sliding on the pre-cut plane. (Online version in colour.)

If the rock permeability is high, even for a range of fluid viscosities, the fluid pressure is expected to be transmitted throughout all the rock pores and onto the fault plane. This is the ‘classic’ case of the effect of pore pressure on rock failure, in which the whole Mohr circle is shifted to the left (lower resolved normal stress, *σ*_n_, values) as a result of increasing pore pressure. Anticipated behaviour for a 45° sawcut is shown in figure 6. A loading path at constant confining pressure (50 MPa) and zero pore pressure is shown as an expanding series of Mohr circles. The locus of points representing shear stress/normal stress on the fault plane joins the points of maximum shear stress and is steeper than the frictional sliding criterion. At the point of initiation of sliding, pore pressure can then be progressively raised, reducing effective normal stress components and moving the stress circle to the left. In this way the stress conditions on the fault could be made to track the entire frictional sliding line on a Mohr diagram. In figure 6, the rock would be sufficiently strong to resist the formation of a new fault plane.

**Figure 7.**
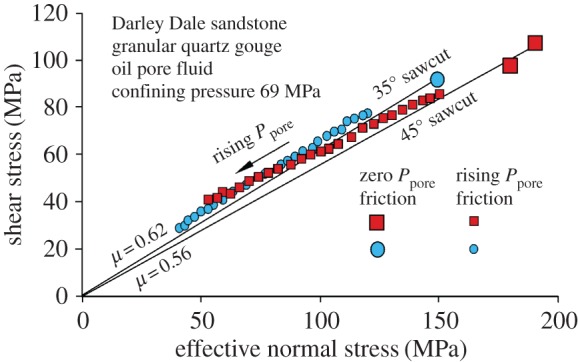
Experimental data (small symbols) that demonstrate offloading of a 35° and a 45° sawcut (samples DDA24 and DDA25, respectively) by sliding as oil pore pressure was progressively increased in Darley Dale sandstone at 69 MPa constant applied confining pressure, in accordance with prediction in figure 6. A thin layer of quartz fault gouge in the slip plane ensured high conductivity. Large symbols show frictional sliding behaviour established during loading without pore pressure. (Online version in colour.)

Experimental results exhibiting this behaviour are shown in figure 7 for the permeable Darley Dale sandstone (oil injection), for sawcuts at 35° and 45° to maximum compressive stress direction. The leftward-declining values of shear stress and effective normal stress (the latter calculated as resolved normal stress minus applied pore pressure) on the fault plane are shown as pore pressure was increased. They follow tracks close to but apparently slightly above the friction sliding criterion established from tests at zero pore pressure, which may mean that the fluid pressure was not completely uniform over the fault plane.

**Figure 8.**
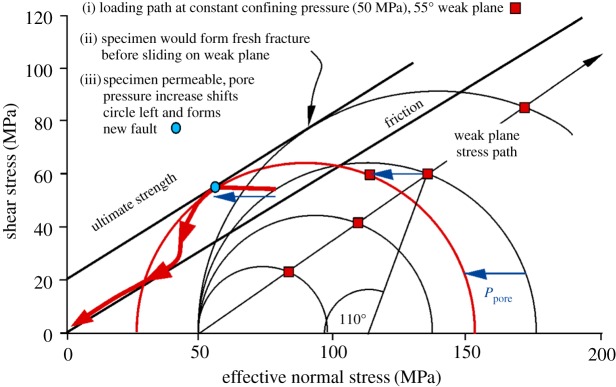
Predicted behaviour of permeable Darley Dale sandstone bearing a 55° pre-cut plane, loaded under constant 50 MPa pore pressure. The stress path for the weak plane is subparallel to the friction sliding line. Raising pore pressure (leftward arrows) for the stress circle shown moves the circle leftward, resulting in formation of a fresh fault before the condition for slip on the weak plane is met. The sample then offloads by sliding on the new fault plane as pore pressure is progressively increased. (Online version in colour.)

Pre-cut orientations of 35° and 45° to the cylinder axis were sufficiently favourable for slip that fresh faulting was not initiated by displacing the Mohr circle to the left, despite the relatively low cohesive strength of Darley Dale sandstone. Figure 8 shows the expected behaviour when fluid pressure is increased when the pre-cut is unfavourably oriented (55°). The displaced Mohr circle intersects the fresh faulting criterion before the slip condition on the sawcut is reached. If this happens, it tells us that the whole Mohr circle has been displaced. Continued increase in fluid pressure should offload the system via slip on the new fault surface, rather than the pre-existing one.

**Figure 9.**
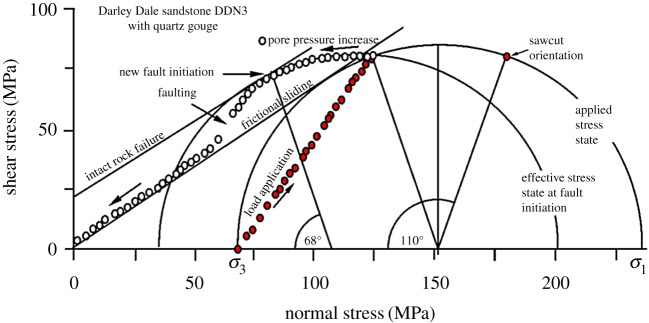
Experimental realization of the predictions of figure 8 (test DDN3). Increasing oil pore pressure caused formation of a fresh fault at 34° to the maximum principal stress direction, before slip conditions on the weak plane were produced. The rock unloaded by slip on the new fault, which showed frictional wear grooves while the 55° weak plane did not. (Online version in colour.)

**Figure 10.**
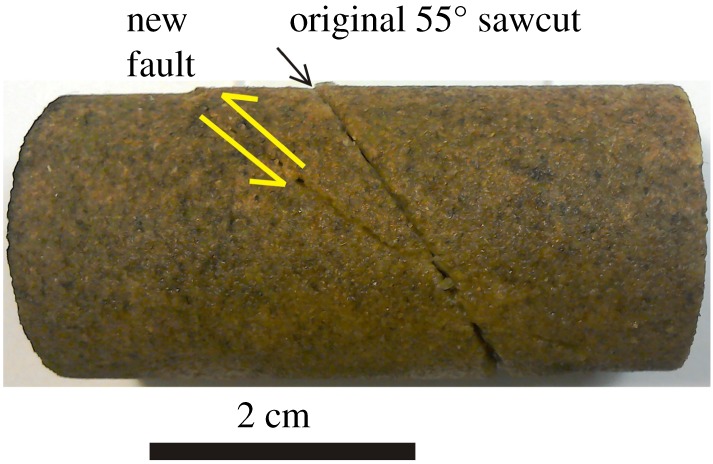
Darley Dale sandstone specimen DDN3 that was deformed in figure 9, showing the new fault plane (labelled showing shear sense) formed at 34° to (horizontal) *σ*_1_, and no sliding on the original sawcut weak plane at 55° to *σ*_1_ (labelled). (Online version in colour.)

Darley Dale sandstone was observed to exhibit this behaviour (sample DDN3) with a 55° sawcut (oil injection, figure 9). A fresh fault formed at 34° to maximum principal stress and the specimen offloaded by slip along this plane as pore pressure continued to be increased. There was no slip along the pre-cut plane. Figure 10 shows the production of a fresh fault in this way.

### (b) Case of rock of low matrix permeability, but conductive fault plane

If the matrix conductance is low but that of the fault plane remains high, pressurized pore fluid can be injected only along the fault plane via the axial hole in the specimen, reducing the effective normal stress 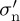 on the fault plane below the total *σ*_n_ applied from the far field.

**Figure 11.**
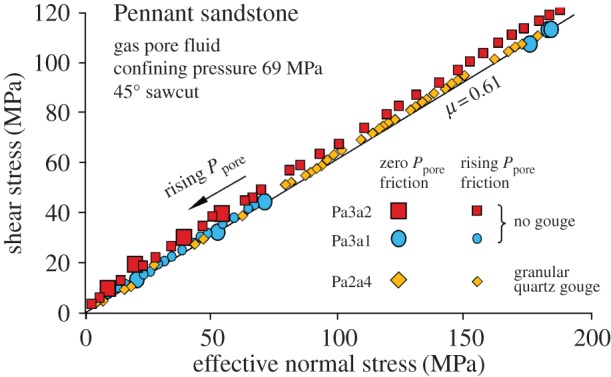
Pennant sandstone (impermeable) showing progressive offloading by slip along 45° sawcuts as a result of gas injection into the fault plane for the specimen numbers indicated. Total confining pressure = 69 MPa. The gas cannot permeate the whole sample but it can penetrate into the sawcut. Large symbols show tests in which a static pore pressure was applied before loading. In all cases the law of effective stress is followed for the fault plane, but the Mohr circle is not shifted leftward. (Online version in colour.)

Figure 11 shows offloading of this rock by gas injection-induced slip on a 45° sawcut both with and without fault gouge in the slip plane. Gas injection was used in the experiments to ensure flow into the fault plane while flow into the rock matrix was still inhibited, although the hydraulic conductivity of a gouge-bearing fault plane would still be sufficiently high even if oil injection were to be used (see below). Friction measured by offloading during gas injection corresponds closely to the friction coefficient measured in tests without pore pressure over a range of normal stress values, hence the effective stress law applies.

In case (b) raising the pore pressure is not expected to shift the whole Mohr circle to the left, only the shear stress/normal stress combination that applies on the fault plane. In this way it should be possible to bring about fault slip on an unfavourably oriented fault plane where, for example, maximum principal stress is at a high angle to the fault plane. This happens without shifting the left side of the Mohr circle into the tensile failure regime, where hydraulic fracture might occur, and without precipitating the formation of a fresh, optimally oriented fault surface. This is the crux of the widely discussed point concerning the role of pore pressure on promotion of slip on unfavourably oriented fault planes in nature, e.g. low-angle extensional detachments and faults such as the San Andreas Fault [34–37].

**Figure 12.**
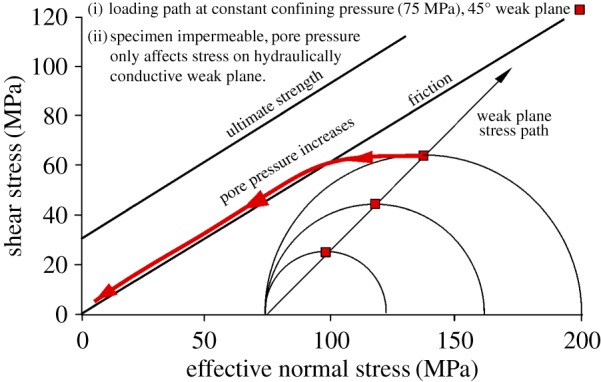
Predicted behaviour of impermeable Pennant sandstone bearing a 45° hydraulically conductive sawcut, loaded from a confining pressure of 75 MPa, then with pore pressure increased from a point below the friction sliding line. The load path for effective normal and shear stresses on the weak plane is shown. The rock offloads along the friction sliding line in accordance with the effective stress law, but the Mohr circle is not displaced to the left. (Online version in colour.)

As the effective stress state on the fault plane is displaced leftward, we expect it to intersect the frictional sliding criterion, causing slip and hence reduction of the shear stress. As previously, continued raising of the fluid pressure should cause the effective stress state to track the sliding criterion down to progressively lower resolved shear and normal stresses (figure 12). If fluid pressure is less than fully effective, for example, if there are poorly conductive patches on the fault plane, this is expected to cause an apparent deviation of the stress state to the left of the friction sliding line.

**Figure 13.**
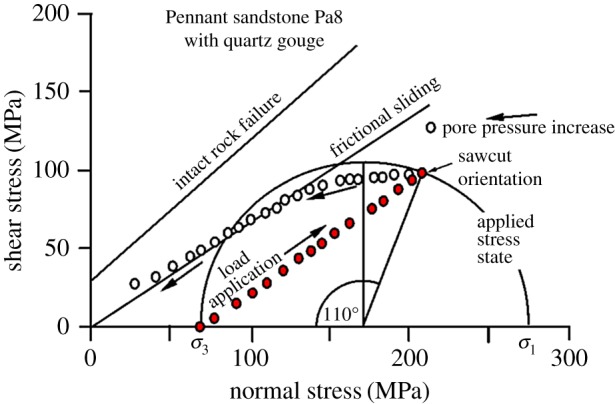
Experimental realization of the predicted behaviour shown in figure 12, but with a sawcut at 55° to *σ*_1_ and a constant total confining pressure of 69 MPa (test Pa8). A quartz gouge layer was placed in the fault plane to ensure permeability to the oil pore fluid. (Online version in colour.)

Figure 13 demonstrates the above predictions for oil injection into an unfavourably oriented (55°) fault plane in Pennant sandstone (e.g. test Pa8), with quartz gouge to enhance the conductivity of the fault plane itself. The initial loading path (combination of normal and shear stress resolved on the weak plane) is subparallel to the sliding criterion, hence sliding would not normally occur on this weak plane before a fresh fault forms due to progressive expansion of the Mohr circle. However, by raising fluid pressure, sliding was initiated on the weak plane, and the system was offloaded by slip on the weak plane as evidenced by frictional wear grooves on the slip plane. No fresh fault was produced, as would happen if the whole Mohr circle were displaced, and no hydraulic fracturing was produced.

### (c) Case of fluid injection into an impermeable host rock with a poorly conductive fault

**Figure 14.**
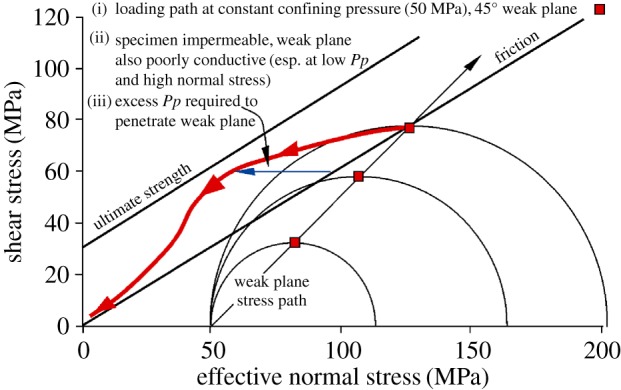
Predicted behaviour of impermeable Pennant sandstone bearing a 45° sawcut, initially hydraulically non-conductive, loaded from a confining pressure of 50 MPa, then with pore pressure increased from the point of initiation of sliding. The expected load path for effective normal and shear stresses on the weak plane requires overpressurization (horizontal leftward arrow) to force the fluid into the fault plane in order to bring about offloading via slip on the weak plane. (Online version in colour.)

In contrast with the case of a granular gouge-bearing fault, Pennant sandstone with no gouge in the fault plane is expected to be poorly conductive for oil injection. In the time scale of an experiment, higher fluid pressures should, therefore, be required to inject the oil, and this would be expected to cause apparent deviations from the effective stress law, as illustrated in the hypothesis shown in figure 14. The measured injection pressure would exceed the true effective pressure inside the fault plane. The latter would be expected to accord with the effective stress law. The difference between the injection fluid pressure and the fluid pressure actually acting in the fault plane can be termed the injection overpressure.

**Figure 15.**
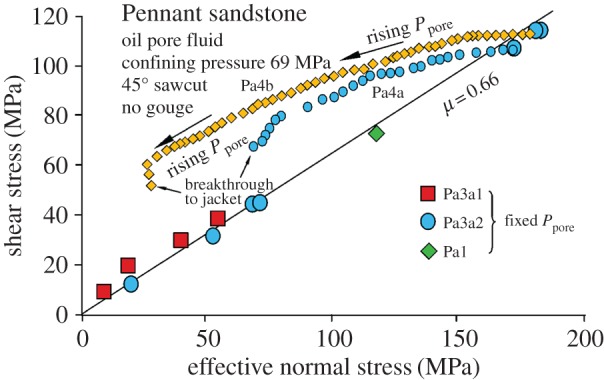
Experimental realization of the predicted behaviour shown in figure 14, for Pennant sandstone with a 45° sawcut, oil pore fluid and no gouge, to ensure poor initial hydraulic conductivity of the fault (tests Pa4a and Pa4b). Overpressure of the injected oil (small symbols, leftward deviation from the friction line) is required to make it inject into the fault plane in the time scale of the experiment. Each test is terminated when the overpressured oil breaks through to the outer surface of the specimen and starts to inflate the jacket. The large symbols show tests in which a fixed pore pressure was applied before loading. These follow the law of effective stress. (Online version in colour.)

**Figure 16.**
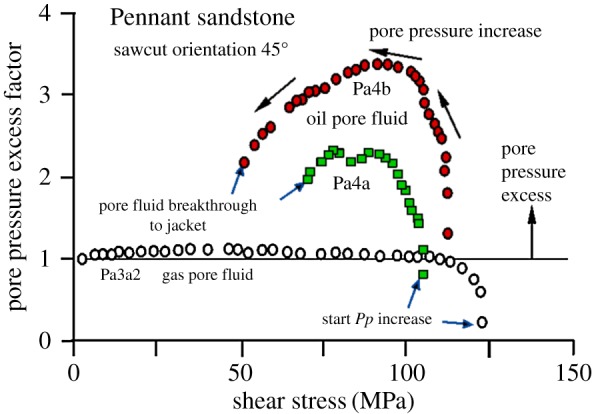
For progressive fluid injection into a low hydraulic conductivity fault (Pennant sandstone without gouge layer), shear stress decreases from its initial value (samples Pa4a and Pa4b). For gas injection (sample Pa3a2) there is no excess pressure required and the sample offloads in accordance with the law of effective stress (constant pore pressure excess factor = 1). For oil injection, excess pressure is needed relative to that required to offload the rock in accordance with the effective stress law (pore pressure excess factor > 1). After initial excess pressure increase it begins to fall, perhaps through slip plus dilatancy-induced fault permeability increase, until the test is terminated by fluid breakthrough to the specimen jacket. (Online version in colour.)

Figure 15 shows experimental results for samples of Pennant sandstone with 45° sawcuts and no quartz gouge with oil injection. Overpressures were required to initiate sliding in experimental sawcut faults (tests Pa4a and Pa4b). As the faults are progressively offloaded, the amount of required fluid overpressure decreases, probably because the decreasing effective normal and shear stresses on the fault, coupled with possible slip dilatancy, increase its hydraulic conductivity. This is illustrated in figure 16, in which the pore pressure excess factor *Px*, defined as the ratio of the applied pore pressure to the pore pressure in the fault plane that corresponds to the effective stress law being followed (*Px* = 1), is plotted versus shear stress. *Px* = 1 for gas injection, when the effective stress law is demonstrably followed. In these tests, pore pressure injection is terminated when the fluid breaks through to the outside cylindrical surface of the specimen, when it becomes buffered by the confining pressure.

When tests with constant pore pressures are carried out, in which fixed pore pressure is applied before loading, the rock follows the effective stress law rather precisely (figure 15). These results demonstrate that it is not easy to inject fluids into shear-oriented faults and cracks when under load, but becomes easier when such planes are filled with granular gouge, or as normal and shear stresses are decreased, or if slippage induces local dilatancy.

The requirement for pore pressure excess (overpressure) in these injection tests implies either that in the time scale of the experiments excess fluid pressure is required to force fluid into the fault plane where it intersects the injection hole, and/or that the fluid ‘fingers’ its way into the slip plane, only partially supporting the applied normal load, unless sufficient excess pressure is applied in the fingers.

Thus the effective normal stress 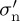 is given by
4.1
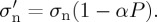


**Figure 17.**
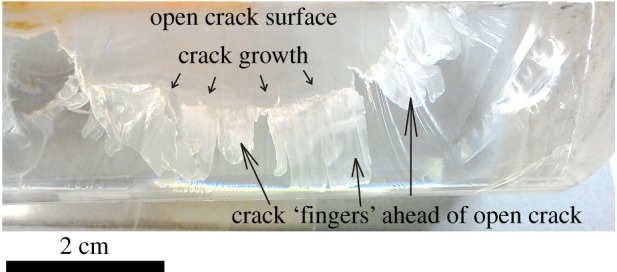
Injection fluid ‘fingering’ (labelled) beyond the downward-growing tip of a demonstration hydraulic fracture in a PMMA (Perspex) block. View is onto the plane of the fracture. (Online version in colour.)

Here, *α* represents the fraction of the fault area that is flooded with fluid injected at pressure *P* at any instant, and it cannot be presumed always to be unity. There is reason to believe [38,39] that fingering of fluid pathways into a stressed plane commonly occurs, so that not all the externally applied normal stress may be reduced by the amount of the fluid pressure, and an excess pressure in the fluid-wetted areas is required to overcome the normal load sufficiently to cause slip. Figure 17 shows fluid fingering of a fluid injected into a transparent polymethyl methacrylate (PMMA) block used to demonstrate hydraulic fracture. In none of the experiments described thus far were seismogenic stress drops observed during offloading, although episodes of relatively rapid but stable sliding were seen.

### (d) Case of fluid injection into a stressed fault via hydraulic fracture

**Figure 18.**
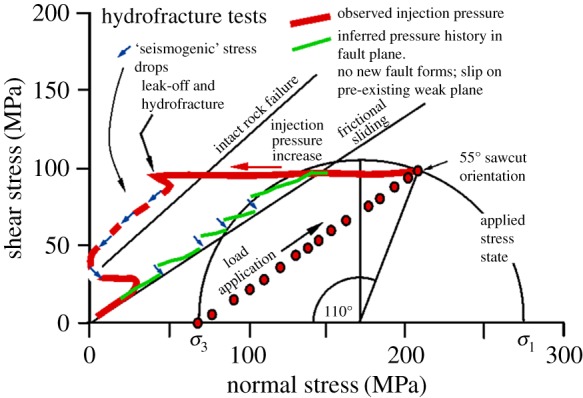
Predicted behaviour of low-permeability Pennant sandstone when a hydraulic fracture must form to link the fluid injection point to the 55° oriented fault plane (figure 5*c*). At the end of the initial loading path oil is injected until the pressure is more than twice the confining pressure. Leak-off and hydrofracture growth then supply overpressured oil to the fault plane, which offloads in a series of seismogenic, stick–slip stress drops (short arrows) until breakthrough to the specimen jacket occurs. In the fault plane itself, effective stress probably follows approximately the friction line as indicated, but punctuated by the stress drops. (Online version in colour.)

Having to produce a hydraulic fracture before a fluid can be injected into a fault potentially allows a large fluid overpressure to be built that is then suddenly discharged into a fault plane via the hydraulic fracture. Figure 18 shows predicted behaviour of the externally measured fluid pressure evolution in this case, and the interpreted fluid pressure pathway in the fault plane.

**Figure 19.**
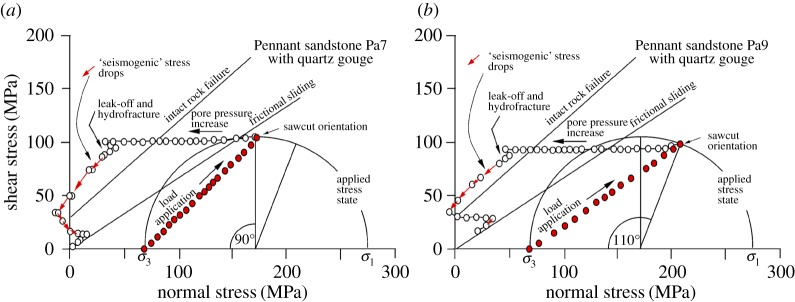
Experimental realization of the behaviour predicted in figure 18 for Pennant sandstone with (*a*) 45° and (*b*) 55° sawcut orientations (tests Pa7 and Pa9). Initial raising of fluid pressure begins from an applied confining pressure of 69 MPa. Leak-off and hydrofracture eventually supply fluid to the fault plane, but a large injection overpressure (leftward deviation of fluid pressure track from the friction sliding line) is still required. In each case five or six seismogenic stress drops (short arrows) occurred as oil flooded into the fault plane via the hydrofracture. (Online version in colour.)

Samples Pa7 and Pa9 (table 2 and figure 19, 45° and 55° sawcuts, quartz gouge present) demonstrate these effects through experiments. Successful underground hydraulic fracturing from a borehole with a minimum of overpressure is likely to depend on the wallrock being damaged either by explosions from casing perforations or by taking advantage of pre-existing flaws (cracks or joints). The critical crack length for initiation of hydraulic fracture at approximately 100 MPa fluid pressure in brittle rocks is expected to be of the order of 0.5 mm [40,41] and this is the order of expected size of flaws in our experiments. In these experiments the starter flaw was the bottom of a short (4 mm) hole at the top of the specimen (figure 5). In common with most experiments, the total confining pressure was made to be constant at 69 MPa and a shear stress of approximately 100 MPa was applied, resulting in an initial resolved normal stress of approximately 180 MPa on a 45° sawcut and a differential stress of similar magnitude. This shear stress is not sufficient to cause sliding on the pre-cut weak plane, and the Mohr circle is still well below the fresh failure criterion for Pennant sandstone (figure 3). Fluid pressure was applied to the notch at the top of the specimen. About 140 MPa total oil pressure was required to cause hydraulic fracture in each case, about twice the confining pressure on the outer surface of the rock cylinder. The pressure seal around the entry hole at the top of the specimen prevented lateral leakage of injection fluid away from the injection point.

**Figure 20.**
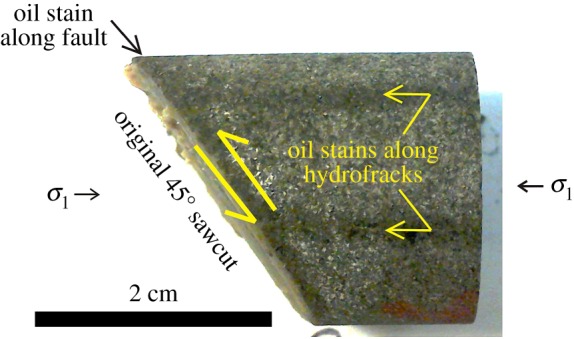
The half of Pennant sandstone specimen Pa9 (55° sawcut) that was connected rightward to the fluid inlet pipe showing traces of two hydrofractures where oil is still seeping onto the cylindrical outer surface (labelled). The rock immediately adjacent to the slip surface is also dark-stained by injected oil. *σ*_1_ is horizontal (indicated). No hydrofracturing penetrated to the lower half of the sample. (Online version in colour.)

The eventual intersection of the hydraulic fractures with the top of the fault plane rapidly injected excess pressure stepwise into the fault plane, causing a time sequence of five or six seismogenic slip events (figure 19). Typically three or four hydraulic fractures were produced, radiating from the fluid injection point, and they could be recognized on the outer surface of the specimen after the test because they continued to seep oil (figure 20, sample Pa9).

The effective pressures plotted on figure 19 are calculated normal stresses on the fault plane minus the fluid pressure applied at the top of the specimen. Thus, they are not the true effective normal stresses on the fault plane, except towards the end of the experiment, when connected pathways between the fluid injection point and the fault plane have become established. At this point the fluid pressure has dropped to become equal to the confining pressure. Any excess fluid volume is able to bulge the jacket away from the specimen and the shear stress on the fault plane falls to zero.

## 5. Discussion

These experiments were carried out at a radically different length scale compared with fluid injection into deep boreholes. Nevertheless, the results provide useful insight into the behaviour of rocks in real fluid injection scenarios. The results show that the relative permeability of the rock matrix compared with the hydraulic conductance of the network of cracks and faults that may exist in the subsurface determines the behaviour of the rock mass for a given fluid viscosity.

When the rock matrix is sufficiently permeable, combined with a sufficiently low injection fluid viscosity, the effective pressure on fault and joint planes is the same as on similarly oriented imaginary planes in the rock mass, and the effective stress law can be simply applied. Increasing pore pressure by injection shifts the whole Mohr circle to the left by an amount equal to the pore pressure. If there is a pre-existing weak plane unfavourably oriented with respect to the principal stresses, for example, at a high angle to *σ*_1_, then the leftward-shifting Mohr circle may intersect the fresh faulting criterion before conditions for slip on the weak plane are attained. If the differential stress is sufficiently low to prevent fresh shear faulting, then hydraulic fracturing may occur as the left side of the Mohr circle is displaced into the tensile field, leading to leak-off of the pore pressure [35]. Thus, in this case raising pore pressure is not a solution to the problem of why slip might be made to occur on unfavourably oriented faults. Note that this simple effective pressure law (with *α* = 1) does not necessarily apply to the effect of fluid pressure on all rock physical properties, such as strength, elastic properties and permeability [42,43].

When the rock matrix is relatively impermeable (on the time scale of fluid injection and subsequent pressure dissipation), for example, in shales, tight sands and some carbonate rocks, dissipation of injection pressure can only take place by flow through a pre-existing network of sufficiently conductive joints and faults, or freshly formed hydraulic fractures. This affects markedly the behaviour of the rock mass. The hydraulic conductivity of planar cracks is expected to be reduced when they are at high angles to *σ*_1_ of the regional stress field because high resolved normal stress along shear-oriented weak planes tends to clamp them shut [44]. Results previously reported by Barton *et al*. [45,46] suggest that this may not be true if they become critically stressed to the point of frictional sliding. Dilatation of the fault plane during sliding is expected to increase hydraulic conductivity, as demonstrated by Guglielmi *et al*. [18]. Hydraulic conductivity of cracks and faults may also be affected by what is in them. The presence of a sufficiently porous granular gouge in fault planes may render them relatively hydraulically conductive, but on the other hand, a very fine-grained granular fault gouge, a clay-bearing gouge or formation of hydrothermal cements would be expected to render faults and cracks non-conductive [44].

It is not unusual in nature for the regional stress field to be unfavourably oriented with respect to pre-existing fault planes but on which slip is known to occur either because they are evidently currently seismogenically active (e.g. San Andreas Fault) [47] or because geological evidence (e.g. low-angle faults in an extended terrane [34,37,48]) shows they slipped while being unfavourably oriented. In such cases, high fluid pressure has often been invoked to permit slip. As we show here, slip can be activated on such unfavourably oriented planes by raising pore pressure on a fault of a typical friction coefficient of 0.65. A low host rock permeability prevents pore fluid from flooding the rock matrix, so that the Mohr circle is not shifted to the left. Instead, only the effective normal stress on the weak plane itself is reduced, eventually precipitating slip.

When additionally the hydraulic conductivity of a pre-existing fault plane is low, excess injection pressure may be required to force fluid into the fault plane. Also, if fluid does not uniformly pressurize the whole fault plane as a result of ‘fingering’, excess fluid pressure in the wetted areas may be needed to reduce the overall effective normal stress.

Guglielmi *et al*. [18] carried out *in situ* mesoscale tests in an underground research laboratory, inducing fault slip by fluid injection and measuring the displacements produced. They demonstrated that most induced movement was aseismic in a rock with a sliding friction coefficient of 0.67 and an intrinsic velocity strengthening characteristic. In this study also, with the exception of the tests involving hydraulic fracture, fault slippage was aseismic and stable as pore fluid was injected. In deep fluid injection via boreholes, it is generally not known what proportion of induced fault movement is aseismic versus seismogenic. The aim of all fluid injection engineering must be to make all induced fault slippages aseismic, and this must be accomplished by careful management of the injection volume and pressure history, coupled with *in situ* monitoring to detect the onset of low-level seismicity [11,17,49].

Hydraulic fractures were used in the present study to apply a sudden, overpressured volume of fluid to the pre-existing fault plane. There was presumably a pressure gradient established to force the fluid from the injection point, where the pressure was measured, through the hydraulic fractures and into the fault plane; therefore, the pressure fluctuations in the fault plane are not known. Nevertheless, this provoked a series of five or six piecemeal fluid pressure drops accompanied by small but audible stress drops (stick–slip sliding), which overrode the normal tendency in these rocks to velocity hardening and stable sliding [50]. We infer that understanding the unsteady injection of fluid into cracks and faults, driven by the stored energy in the overpressured fluid, will be at the heart of developing strategies for managing fluid injection without inducing seismicity. Developing such understanding is probably best done using a combination of modelling and mesoscale *in situ* experiments.

## 6. Conclusion

We carried out experiments on samples of permeable Darley Dale sandstone and impermeable Pennant sandstone bearing pre-existing sawcuts variously inclined to the maximum compressive stress. Fault slip was provoked by increasing injection fluid pressure (gas or liquid).

Injection of fluid into permeable rock followed the law of effective stress; the Mohr circle was displaced leftward until either fresh fault formation occurred or slip on the weak plane occurred according to its orientation. Fluid injection into impermeable rock was focused into the weak plane, reducing the effective pressure in the plane until sliding occurred, but without displacing the effective stress state in the host rock. In both cases, the rock could be offloaded aseismically by slip along the fault plane.

Sudden application of fluid pressure to the weak plane via a hydraulic fracture provoked seismogenic offloading, because of accumulation of a fluid volume subjected to the excess pressure necessary to drive the hydraulic fracture, which overwhelmed the otherwise tendency for stable sliding in these rocks. These results point to the need to develop techniques to manage the rate and volume of fluid injected via deep boreholes, and to avoid the build-up of excess fluid pressures at the point of injection.

## Supplementary Material

Rutter & Hackston supplementary data file.csv

## Data accessibility

Raw data as time series for each experiment reported are provided as the electronic supplementary material.
